# Prescription of benzodiazepines and related drugs in the Psychiatry Department in the Psychiatry department of Tahar Sfar, Mahdia hospital

**DOI:** 10.1192/j.eurpsy.2023.682

**Published:** 2023-07-19

**Authors:** S. Brahim, W. Bouali, M. E. bakhouch, M. Kacem, S. Khouadja, R. Ben Soussia, S. Younes, L. Zarrouk

**Affiliations:** Psychiatry, Hôpital Taher Sfar, Mahdia, Tunisia

## Abstract

**Introduction:**

Benzodiazepines are the most widely prescribed drugs worldwide for insomnia and anxiety disorders. However, few studies have been conducted on the professional practice of these drugs for patients with psychiatric disorders .

**Objectives:**

To describe the prescribing practices of benzodiazepines for patients with psychiatric disorders at the Psychiatry Department of the EPS Taher Sfar Mahdia.

**Methods:**

This is a retrospective study of patients who were admitted for the first time to the psychiatry department of the EPS Taher Sfar in Mahdia and had a prescription of benzodiazepines during their hospitalization.

**Results:**

A total of 234 patients were included in our study. We found that 77.8% of patients on benzodiazepines had a prescription for benzodiazepines for a period of less than 3 months. Secondly, we determined that 66.2% of patients who had a benzodiazepine’s prescription had a taper dose of benzodiazepines before the withdrawal. No patients with contraindications to benzodiazepines had a prescription of these medications. The maximum indicated dosage was respected in 92.3% of the prescriptions. Lorazepam was the most used drug, accounting for 49.1% of prescriptions. Our study showed that 46.2% of prescriptions were for anxiolytic purposes only, 43.2% were for hypnotic purposes only. Our analysis also showed a higher proportion of males in the < 3 months group with 82.9% which is significantly higher than for females. (p=0.004).

Our analytical study concluded that gender (p=0.004), professional status (p=0.014), history of addiction (p=0.003), cannabis use (0.025) were related to the duration of benzodiazepine prescription. We noted that 89.9% (n=71) of patients with a documented history of addiction had been prescribed benzodiazepines for less than 3 months. We were also able to conclude that there were correlations between the duration of prescription and medical and/or surgical history (p=0.002), the molecule prescribed (p=0.0001) as well as the renewal of the prescription (0.0001).

However, we did not find a correlation between the associated psychiatric disorders and the duration of prescription. As well for associated psychotropic drugs and duration of prescription

**Conclusions:**

We can conclude that misuse of benzodiazepines exists, but to a much lesser extent than in the literature. A larger-scale study would be essential to establish a Tunisian overview of benzodiazepine prescription practices.

**Disclosure of Interest:**

None Declared

